# A spiking network model of basal ganglia to study the effect of dopamine medication and STN-DBS during probabilistic learning task

**DOI:** 10.1186/1471-2202-16-S1-P25

**Published:** 2015-12-18

**Authors:** Alekhya Mandali, V Srinivasa Chakravarthy

**Affiliations:** 1 Department of Biotechnology, Bhupat and Mehta School of Biosciences, Chennai, 600036, Tamil Nadu, India

## 

The effect of dopamine medication (L-Dopa and Dopamine (DA) agonists) and Deep Brain Stimulation (DBS) of Sub Thalamic Nucleus (STN) on the cognition of Parkinson's disease (PD) patients has been of interest to neuroscientists, owing to their ability to produce impulsive behavior as a side effect. Interestingly, it has been found that the impulsive decision making in STN-DBS patients has been observed to be quite contrary. As an attempt to understand the aforementioned side effect, we built a spiking network model of basal ganglia (BG) and tested on a probabilistic learning task [1]. BG nuclei such as Globus Pallidus externa (GPe), interna (GPi) and STN were represented as 2D arrays of Izhikevich neurons (50x50 lattice) and the activity of Striatal neurons (D1 and D2 receptor expressing Medium Spiny Neurons) as a Poisson process. Invoking the idea that DA cells in Substantia Nigra par compacta code for reward prediction error, similar to the temporal difference (TD) error term in Reinforcement learning, learning was introduced by updating the cortico-striatal weights using TD error. In the probabilistic learning task, the system was subjected to 3 pairs of stimuli (one at a time) randomly and the goal is to learn to select the most rewarding stimulus (=A) and avoid the punishing one (=B).The task had both training and testing stages where the model had to learn to make an optimal decision. The model's performance was tested on Healthy controls, PD in 'OFF', PD 'ON' (L-Dopa & DA agonists) and finally on STN-DBS in terms of percentage accuracy and Reaction time (RT).

## Results

**Figure 1:**
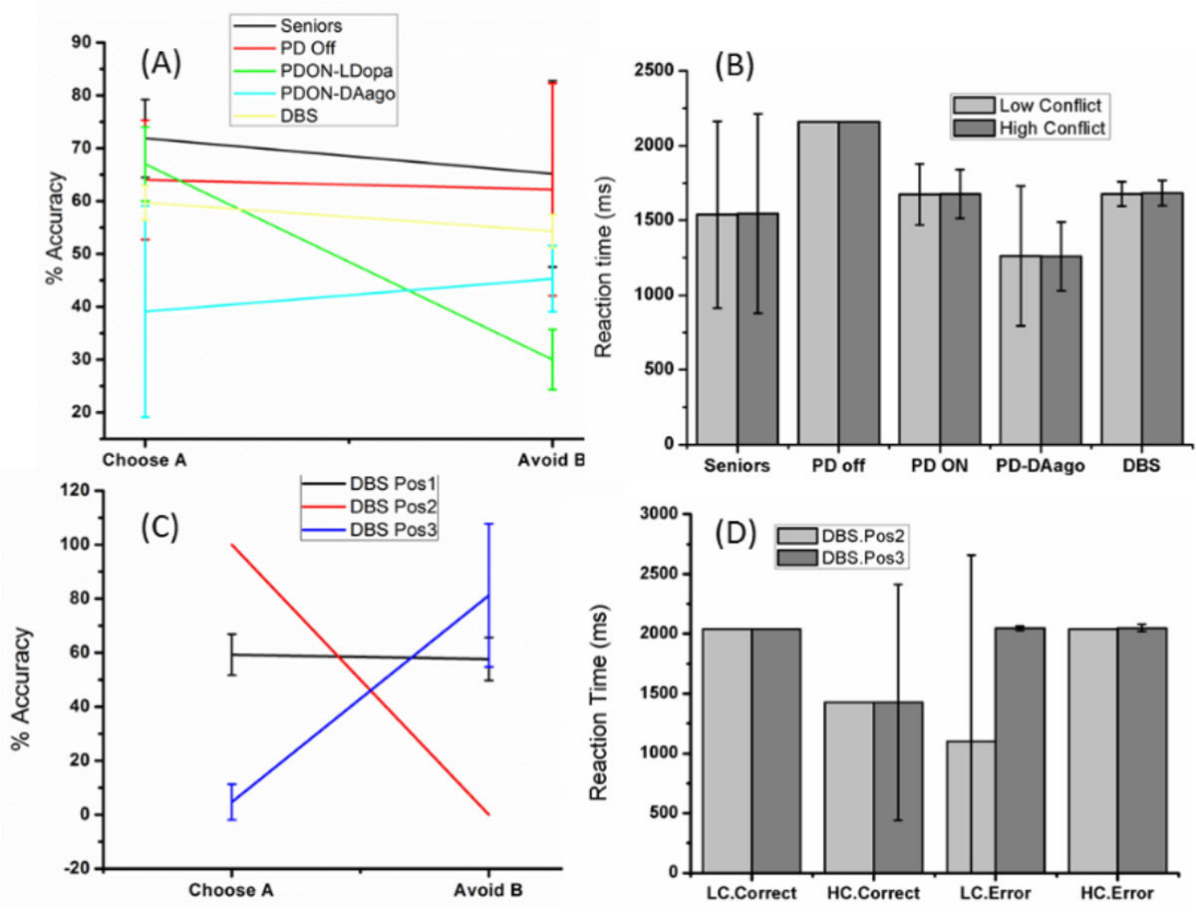
**Percentage Accuracy and Reaction times for various simulated of network model**. (A) % percentage accuracy (B) Reaction Times for Low and High conflict conditions. (C) % Accuracy levels for different positions of DBS electrode in STN (D) and the corresponding Reaction times.

The performance PD-OFF cases showed skewness towards punishment learning (avoiding 'B'), whereas the opposite was observed in PD-ON (L-Dopa) case (fig. 1A). These results are consistent with experimental results which suggest that the low (high) DA in PD-OFF (ON) state leads to higher punishment (reward) sensitivity. Our model results are also consistent with other experimental studies related to DA agonist effects on decision making. The lowest choice reaction times for PD-DA agonist condition (Fig 1B) (among all other cases) indicates impulsivity leading to suboptimal action selection observed in decreased % Accuracy levels (Fig. 1A). The effect of DBS electrode position on RT and % accuracy levels (Fig. 1C & 1D) were found also to be interesting, where a change in electrode position made the model performance to switch from punishment learning to reward learning. These results suggest that the DBS electrode position might play a major role in inducing impulsivity in the PD patients.
